# Placental Growth Factor as a Predictor of Adverse Maternal Outcomes in Patients With Hypertensive Disorders of Pregnancy: A Prospective Observational Study

**DOI:** 10.7759/cureus.83285

**Published:** 2025-05-01

**Authors:** Rakshitha Raghavendra, Shailaja Bidri, Rajasri G Yaliwal, Neelamma Patil, Aruna Biradar, Preeti S Malapure

**Affiliations:** 1 Obstetrics and Gynaecology, Shri B. M. Patil Medical College Hospital and Research Centre, BLDE (Deemed to be University), Vijayapura, IND

**Keywords:** hypertension, hypertensive disorders of pregnancy (hdp), maternal outcomes, placental growth factor, preterm

## Abstract

Introduction: Hypertensive disorders of pregnancy (HDP) are among the leading causes of maternal and perinatal morbidity and mortality, primarily due to angiogenic imbalance and impaired vascular remodeling, resulting in elevated blood pressure and multiorgan involvement. Early detection of these conditions is crucial to improve outcomes. This study evaluates the role of serum placental growth factor (PLGF) as a biomarker for the severity of HDP and its association with maternal and neonatal outcomes. The objective of the study is to establish the diagnostic efficacy of serum PLGF in predicting adverse maternal outcomes in women diagnosed with HDP.

Materials and methods: A prospective observational study was conducted at the Department of Obstetrics and Gynaecology, B.L.D.E. (Deemed to be University), Shri B.M. Patil Medical College Hospital and Research Centre, Vijayapura. A total of 164 pregnant women diagnosed with HDP were enrolled after obtaining informed consent. Peripheral venous blood samples were collected on admission for serum PLGF testing using ELISA (enzyme-linked immunosorbent assay). Participants were monitored for maternal and perinatal outcomes (as per the American College of Obstetricians and Gynecologists (ACOG) guidelines), and correlations between PLGF levels and disease severity were analyzed.

Results: Of 164 hypertensive women, 91 (55.5%) had normal and 73 (45.5%) had low PLGF values. Significant associations (p < 0.05) were found between low PLGF and clinical/laboratory variables, including mode of delivery, imminent signs, hypertensive category, urine albumin, amniotic fluid volume, adverse maternal complications, and neonatal outcomes. A negative correlation was noted between PLGF and both systolic and diastolic blood pressures. The sensitivity and negative predictive values of PLGF were 83.5% and 72.1%, respectively.

Conclusion: Serum PLGF levels are significantly associated with the severity of HDP and with adverse maternal and perinatal outcomes.

## Introduction

Pregnancy-induced hypertension (PIH) remains one of the leading contributors to maternal and neonatal morbidity and mortality, affecting approximately 5% to 8% of all pregnancies [1]. Hypertensive disorders of pregnancy (HDPs), which encompass preeclampsia, gestational hypertension, and chronic hypertension, are associated with serious obstetric complications including fetal growth restriction, stillbirth, preterm birth, and neonatal morbidity [2]. The clinical presentation of HDPs is notably heterogeneous, making accurate prediction and timely management a considerable challenge.

Despite its significant impact on perinatal outcomes, hypertension alone accounts for only about 20% of adverse outcomes in preeclampsia [3]. Moreover, up to 10% of affected women may exhibit minimal or no clinical symptoms even in the presence of severe disease, further complicating early diagnosis and risk stratification [3]. The limited predictive capacity of current diagnostic approaches has led to unnecessary interventions, prolonged hospitalizations, and an increase in iatrogenic preterm deliveries, thereby inflating healthcare costs without necessarily improving outcomes [2,3].

Over the past 15 years, research into placental biomarkers has gained prominence in the quest to better understand and manage preeclampsia. A major focus has been on angiogenic factors such as placental growth factor (PLGF) and soluble fms-like tyrosine kinase-1 (sFlt-1) [4]. PLGF, a proangiogenic protein belonging to the vascular endothelial growth factor (VEGF) family, normally rises throughout pregnancy and peaks between 26 and 30 weeks of gestation [5,6]. Following this peak, levels naturally decline as the pregnancy progresses toward term. However, in preeclamptic pregnancies, PLGF levels are markedly reduced, often preceding the clinical onset of the disease by several weeks [6,7]. These reductions reflect underlying placental dysfunction and have emerged as a potential early indicator of adverse pregnancy outcomes.

Integrating novel angiogenic biomarkers such as PLGF into clinical practice may significantly improve the detection and management of hypertensive disorders in pregnancy. By enabling earlier and more accurate identification of at-risk pregnancies, PLGF measurement could help reduce unnecessary interventions and optimize care delivery.

This study aims to assess the clinical utility of serum PLGF as a predictive marker for adverse maternal and neonatal outcomes in hypertensive pregnancies. By correlating PLGF levels with disease severity and complications, the study seeks to support the integration of this biomarker into routine risk assessment strategies. Early detection of complications using PLGF could potentially alleviate the burden of recurrent hospital admissions, intensive care needs, and the associated healthcare costs, ultimately improving outcomes for both mother and child.

## Materials and methods

This prospective observational study was conducted at the Department of Obstetrics and Gynecology, BLDE (Deemed to be University), Shri B.M. Patil Medical College, Hospital & Research Centre, Vijayapura. The study was approved by the Institutional Ethics Committee (BLDE(DU)/IEC/894/2022-23) and adhered to the ethical standards outlined in the Declaration of Helsinki. All eligible participants were provided with complete information regarding the study, and written informed consent was obtained prior to enrollment. The hospital's status as a tertiary care referral center offered an appropriate clinical environment for evaluating hypertensive disorders in pregnancy and assessing relevant biomarkers.

The study was carried out over a period extending from April 2023 to December 2024. A total of 164 pregnant women were enrolled after fulfilling specific inclusion criteria. The participants were followed during the course of their hospital admission. Data collection included demographic details, clinical parameters, laboratory investigations, ultrasound findings, and sequential clinical events. Blood pressure values recorded at the time of admission were used for analysis prior to any intervention. Peripheral venous blood samples were collected from each participant at admission and subjected to PLGF analysis using an enzyme-linked immunosorbent assay (ELISA) method.

Inclusion criteria comprised pregnant women aged 18 to 45 years, between 28 and 40 weeks of gestation, with a live singleton pregnancy, and diagnosed with any of the HDP as per the American College of Obstetricians and Gynecologists (ACOG) guidelines [8]. The diagnostic categories included preeclampsia, chronic hypertension, chronic hypertension with superimposed preeclampsia, and gestational hypertension. Exclusion criteria were the presence of eclampsia at presentation, multiple pregnancies, intrauterine fetal demise, or known congenital anomalies in the fetus.

The required sample size was calculated to be a minimum of 133, based on an estimated prevalence of low PLGF levels of 66.7%, with 95% confidence and an 8% margin of error. The formula used was $n = Z&sup2; &times; p &times; q / d&sup2;$, where Z is the standard normal variate, p is the assumed proportion, $q = 100 - p$, and d is the acceptable error margin.

PLGF levels were measured using a sandwich ELISA method. The kit used was specific for human PGF detection in serum and was based on pre-coated antibody plates. Upon adding the patient serum, PLGF in the sample bound to the immobilized antibody. A biotinylated PGF detection antibody and streptavidin-HRP were added sequentially. Following a series of incubation and washing steps, a substrate solution was introduced, producing a color change in proportion to the amount of PGF present. The reaction was stopped with an acidic solution, and absorbance was measured at 450 nm.

PLGF values were categorized into two groups based on TRIAGE recommendations: low PLGF (<100 pg/mL) and normal PLGF (≥100 pg/mL). These cutoff values were used to analyze associations with maternal and neonatal outcomes. Maternal complications monitored included eclampsia, HELLP (hemolysis, elevated liver enzymes, and low platelets) syndrome, pulmonary edema, placental abruption, requirement of a third antihypertensive agent, and other severe conditions such as acute renal failure, myocardial infarction, hypertensive encephalopathy, cortical blindness, retinal detachment, stroke, disseminated intravascular coagulation (DIC), thrombotic microangiopathies, acute fatty liver of pregnancy, liver hematoma, or rupture, and maternal death. The diagnostic criteria for these complications were based on clinical diagnoses made by the treating obstetric, medical, and critical care teams, following institutional or national guidelines.

The perinatal outcomes assessed were gestational age at delivery, birth weight, respiratory distress within 24 hours of birth, NICU admission and its indication, and neonatal death. These outcomes were then analyzed in relation to the PLGF levels.

**Figure 1:**
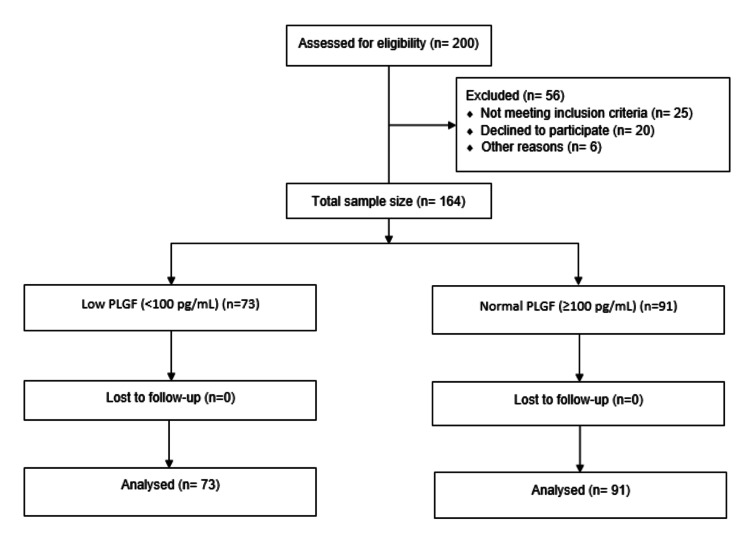
Flowchart of the Study PLGF: Placental Growth Factor

Statistical analysis was performed using IBM SPSS Statistics for Windows, Version 26 (Released 2019; IBM Corp., Armonk, New York). Descriptive statistics were used to summarize the baseline characteristics of the study population. Categorical variables were expressed as frequencies and percentages, while continuous variables were presented as means with standard deviations. The chi-square test was used to evaluate associations between categorical variables. A p-value of <0.05 was considered statistically significant (Figure 1).

## Results

Among 164 antenatal women studied, the distribution of age groups showed that the majority in both normal and low PLGF groups were between 21 and 30 years, with no statistically significant difference observed (p=0.11). Parity types were also comparable between the two groups without a significant association (p=0.55). However, significant variation was noted in the mode of delivery; preterm LSCS (lower segment cesarean section) was more frequent in the low PLGF group (42.5%) compared to the normal PLGF group (24.2%), with a p-value of 0.05. Imminent signs were observed significantly more in the low PLGF group (39.7%) than in the normal group (19.8%), indicating a strong association (p=0.005) (Table 1).

**Table 1:** Demographic and Clinical Characteristics by PLGF Level Frequency and percentages were calculated. A chi-square test was applied, and a p-value of less than 0.05 was considered significant. LSCS: Lower Segment Cesarean Section; VD: Vaginal Delivery; PLGF: Placental Growth Factor

Variables	Normal PLGF (N=91)	Low PLGF (N=73)	Chi-Square Value	p-value
Age (Years)				
15–20	14 (15.4%)	11 (15.1%)	5.965	0.11
21–30	69 (75.8%)	46 (63.0%)		
31–40	7 (7.7%)	15 (20.5%)		
41–45	1 (1.1%)	1 (1.4%)		
Parity Type				
Primigravida	43 (47.3%)	32 (43.8%)	1.178	0.55
Multigravida	37 (40.7%)	35 (47.9%)		
Primipara	11 (12.1%)	6 (8.2%)		
Mode of Delivery				
Preterm LSCS (PLSCS)	22 (24.2%)	31 (42.5%)	10.879	0.05
Full-Term LSCS	50 (54.9%)	34 (46.6%)		
Full-Term Vaginal	13 (14.3%)	3 (4.1%)		
Preterm Vaginal	4 (4.4%)	3 (4.1%)		
Assisted VD	2 (2.2%)	0 (0%)		
Undelivered (Death)	0 (0%)	2 (2.7%)		
Imminent Signs				
Yes	18 (19.8%)	29 (39.7%)	7.882	0.005
No	73 (80.2%)	44 (60.3%)		

Urine albumin levels were significantly elevated in the low PLGF group (p=0.006), with a higher percentage showing proteinuria (+ and above) compared to those with normal PLGF. Oligohydramnios and anhydramnios were more prevalent in the low PLGF group (20.5%) than in the normal PLGF group (8.8%) (p=0.05). Although Doppler abnormalities, including IUGR (intrauterine growth restriction) and uteroplacental insufficiency, were more frequent in the low PLGF group, the difference did not achieve statistical significance (p=0.172) (Table 2).

**Table 2:** Distribution of Laboratory and Ultrasound Parameters with PLGF Levels (N=164) Frequency and percentages were calculated. A chi-square test was applied, and a p-value of less than 0.05 was considered significant. The symbols +, ++, +++, and ++++ in the context of urine albumin represent increasing levels of albumin (protein) present in the urine, which is a sign of proteinuria. + (1+): Mild proteinuria (approximately 30 mg/dL); ++ (2+): moderate proteinuria (approximately 100 mg/dL); +++ (3+): marked proteinuria (approximately 300 mg/dL); ++++ (4+): severe proteinuria (≥1000 mg/dL or more). Trace: A very small amount of albumin may be considered within normal limits or borderline; Nil: no albumin detected in the urine. IUGR: Intrauterine Growth Restriction; SGA: Small for Gestational Age; UPI: Uteroplacental Insufficiency

Variables	Normal PLGF (N=91)	Low PLGF (N=73)	Chi-Square Value	p-value
Urine Albumin				
Nil	27 (29.7%)	11 (15.1%)	16.499	0.006
Trace	7 (7.7%)	6 (8.2%)		
+	41 (45.1%)	38 (52.1%)		
++	9 (9.9%)	11 (15.1%)		
+++	7 (7.7%)	5 (6.8%)		
++++	0 (0.0%)	1 (1.4%)		
Amniotic Fluid Index				
Normal	83 (91.2%)	58 (79.5%)	6.111	0.05
Oligohydramnios	8 (8.8%)	13 (17.8%)		
Anhydramnios	0 (0.0%)	2 (2.7%)		
Doppler Study				
Normal	86 (94.5%)	60 (82.2%)	9.030	0.172
Grade I IUGR	1 (1.1%)	4 (5.5%)		
Grade I IUGR + UPI	1 (1.1%)	4 (5.5%)		
Grade II IUGR + UPI	1 (1.1%)	2 (2.7%)		
Grade II IUGR	0 (0.0%)	1 (1.4%)		
SGA	0 (0.0%)	1 (1.4%)		
Uteroplacental Insufficiency (UPI)	2 (2.2%)	1 (1.4%)		

Antepartum complications occurred more frequently in the low PLGF group (20.6%), with higher rates of pulmonary edema (8.2%) and HELLP syndrome (5.5%), which were significantly associated with low PLGF levels (p=0.04). Intrapartum complications such as ascites and abruptio were also more common in the low PLGF group (11%) compared to the normal PLGF group (1.1%), with statistical significance (p=0.03). Postpartum complications, including pulmonary edema, HELLP, and the need for a third antihypertensive agent, were more frequent in the low PLGF group; however, the association was not statistically significant (p=0.15) (Table 3).

**Table 3:** Distribution of Maternal Complications with PLGF Levels (N=164) Frequency and percentages were calculated. Fisher's exact test was applied, and a p-value of less than 0.05 was considered significant. HELLP: Hemolysis, Elevated Liver Enzymes, and Low Platelet Count; PPH: Postpartum Hemorrhage; AKI: Acute Kidney Injury

Maternal Complications	Normal PLGF (N=91)	Low PLGF (N=73)	Statistical Value	p-value
Antepartum Complications				
Pulmonary Edema	2 (2.2%)	6 (8.2%)	11.984	0.04
Third Antihypertensive Agent	0 (0.0%)	2 (2.7%)		
Grade 2 Retinopathy	1 (1.1%)	0 (0.0%)		
HELLP Syndrome	1 (1.1%)	4 (5.5%)		
Antepartum Hemorrhage	0 (0.0%)	1 (1.4%)		
Thrombocytopenia	2 (2.2%)	1 (1.4%)		
Maternal Death	0 (0.0%)	1 (1.4%)		
No Complication	85 (93.4%)	58 (79.4%)		
Intrapartum Complications				
Ascites	1 (1.1%)	7 (9.6%)	11.825	0.03
Abruptio + Ascites	0 (0.0%)	1 (1.4%)		
Obstructed Labor	0 (0.0%)	1 (1.4%)		
Maternal Death	0 (0.0%)	1 (1.4%)		
No Complication	90 (98.9%)	62 (84.9%)		
Not Applicable	0 (0.0%)	1 (1.4%)		
Postpartum Complications				
Renal Parenchymal Disease	1 (1.1%)	0 (0.0%)	14.371	0.15
Thrombocytopenia	3 (3.3%)	0 (0.0%)		
Third Antihypertensive Agent	2 (2.2%)	4 (5.5%)		
HELLP Syndrome	1 (1.1%)	3 (4.1%)		
Secondary Suturing	2 (2.2%)	0 (0.0%)		
PPH	1 (1.1%)	1 (1.4%)		
Pulmonary Edema	1 (1.1%)	3 (4.1%)		
Postpartum Eclampsia	0 (0.0%)	1 (1.4%)		
AKI + Hepatitis	0 (0.0%)	1 (1.4%)		
No Complication	80 (87.9%)	58 (79.5%)		
Not Applicable	0 (0.0%)	2 (2.7%)		

NICU admissions were higher among neonates born to mothers with low PLGF (38.4%) compared to normal PLGF (26.4%), and this association was statistically significant (p=0.05). Neonatal mortality was reported exclusively in the low PLGF group (5.5%), along with stillbirths (5.5%), while all neonates in the normal PLGF group survived. Mean birth weight was significantly lower in the low PLGF group (2283.49 ± 784.86 g) than in the normal group (2593.07 ± 554.92 g) with p=0.004. Gestational age also differed significantly, with a greater proportion of very preterm births (<32 weeks) in the low PLGF group (9.6%) compared to the normal group (1.1%) (p=0.04) (Table 4).

**Table 4:** Distribution of Neonatal Outcomes with PLGF Levels (N=164) Frequency and percentages were calculated. A chi-square test was applied, and a p-value of less than 0.05 was considered significant. PLGF: Placental Growth Factor; NICU: Neonatal Intensive Care Unit; SD: Standard Deviation

Neonatal Outcomes	Normal PLGF (N=91)	Low PLGF (N=73)	Chi-Square Value	p-value
NICU Admission at Birth				
Yes	24 (26.4%)	28 (38.4%)	6.313	0.05
No	67 (73.6%)	43 (58.9%)		
Not Applicable (Undelivered)	0 (0.0%)	2 (2.7%)		
Neonatal Status				
Alive	91 (100%)	65 (89.0%)	10.484	0.03
Neonatal Death	0 (0.0%)	4 (5.5%)		
Stillbirth	0 (0.0%)	4 (5.5%)		
Birth Weight				
Mean ± SD (grams)	2593.07 ± 554.92	2283.49 ± 784.86	2.954	0.004
Gestational Age at Birth				
Very Preterm (28–<32 weeks)	1 (1.1%)	7 (9.6%)	10.484	0.04
Moderate to Late Preterm (32–37 weeks)	27 (29.7%)	31 (42.5%)		
Term (>37 weeks)	63 (69.2%)	35 (47.9%)		

A significant association was found between prior history of hypertension and low PLGF levels (p=0.01) (Table 5).

**Table 5:** Association Between Hypertension History and PLGF Level Frequency and percentages were calculated. A chi-square test was applied, and a p-value of less than 0.05 was considered significant. PLGF: Placental Growth Factor

Hypertension Prior to Admission	Normal PLGF (N=91)	Low PLGF (N=73)	Chi-Square Value	p-value
Yes	60 (65.9%)	61 (83.6%)	6.506	0.01
No	31 (34.1%)	12 (16.4%)		

Hypertension history demonstrated a sensitivity of 83.5% and a specificity of 34.1% in predicting low PLGF, with a positive predictive value of 50.4% and a negative predictive value of 72.1%.

Pearson's correlation revealed a significant negative correlation between PLGF levels and both systolic and diastolic blood pressures. PLGF had a correlation coefficient of -0.337 with systolic blood pressure and -0.417 with diastolic blood pressure, both with p-values <0.001, indicating a strong inverse relationship (Table 6).

**Table 6:** Correlation Between Blood Pressure and PLGF Levels Pearson's correlation test was done, and a p-value of less than 0.05 was considered significant. SBP: Systolic Blood Pressure; DBP: Diastolic Blood Pressure

Variables	Pearson's Correlation	p-value
SBP	-.337	<0.001
DBP	-.417	<0.001

## Discussion

The timely diagnosis of HDP is crucial for safeguarding maternal and fetal health. Early identification through clinical signs, risk stratification, and confirmation with reliable biomarkers can significantly reduce complications and associated healthcare costs. Among the various emerging diagnostic tools, PLGF has garnered attention due to its potential role in the pathogenesis of preeclampsia, which is characterized by an imbalance of circulating angiogenic and anti-angiogenic factors. While PLGF testing does not markedly increase the diagnosis rate of preeclampsia, it significantly reduces the time to diagnosis across all thresholds of PLGF concentration [9].

This prospective observational study sought to investigate the relationship between serum PLGF levels and the clinical, demographic, laboratory, ultrasound, and outcome-related parameters in women diagnosed with HDP. The results reinforce the potential role of PLGF as a predictive biomarker.

The mean maternal age among the 164 participants was 25.53, with the majority falling within the 21-30-year age group. Interestingly, maternal comorbidities such as hypothyroidism and gestational diabetes mellitus did not significantly influence PLGF levels in this cohort.

Clinically, the majority of patients with low PLGF underwent cesarean delivery (89%), indicating a high incidence of iatrogenic interventions. This aligns with the findings by Gladstone et al., who reported increased cesarean rates and a six-week reduction in mean gestational age in patients with low PLGF, likely due to placental dysfunction and associated risks [10]. A total of 29 patients with low PLGF presented with imminent symptoms at admission, necessitating expedited delivery. These observations are consistent with the study by McLaughlin et al., which also noted significant associations between low PLGF levels and the need for urgent delivery [11].

Laboratory analysis revealed a significant association between low PLGF and elevated urine albumin levels, reinforcing prior observations by Leaños-Miranda et al., which highlighted the correlation of angiogenic imbalance with proteinuria [12]. From an imaging standpoint, while oligohydramnios showed a significant association with low PLGF, suggesting advanced placental dysfunction, Doppler changes did not, diverging from findings by Molvarec et al. [13]. Nonetheless, the relationship between low PLGF and placental insufficiency remains evident, as discussed in the work by Schlembach et al. [14].

Adverse maternal outcomes were notable in our cohort. Among patients with low PLGF, antepartum complications such as HELLP syndrome and thrombocytopenia were prevalent. This reflects the findings of Lopes Perdigao et al., who reported significant maternal morbidity associated with decreased PLGF levels [15]. Intraoperative findings during cesarean sections, particularly ascites and placental abruption, were also more frequent in patients with low PLGF, as previously reported by Brown et al. [16]. Notably, two maternal deaths were recorded in our study, contrasting with the findings of Lopes Perdigao et al., where no maternal mortality was observed despite other complications [15].

Perinatal outcomes further emphasized the clinical relevance of PLGF. Nearly 38.4% of neonates born to mothers with low PLGF required NICU admission. Additionally, four neonatal deaths and two stillbirths were associated with maternal PLGF deficiency, paralleling the observations of Duhig et al. [9]. The mean birth weight among neonates from the low PLGF group was 2283.49 grams, placing them in the low birth weight category. Furthermore, 38 mothers delivered preterm, with a subset of very preterm births. These outcomes are similar to those reported by McLaughlin et al., reinforcing the predictive capacity of low PLGF for preterm delivery [11].

When examining the diagnostic validity of PLGF, our study demonstrated a sensitivity of 83.5% and a negative predictive value of 72.1%, comparable to other key studies such as those by Benton et al., Das et al., and the Ontario Health [17-19]. Although our specificity was lower, the overall pattern remains consistent with global literature affirming the role of PLGF in predicting HDP-related complications.

Correlation analysis between PLGF and blood pressure measurements showed a negative association, affirming findings from Gbadegesin et al. and Kumar et al. [20,21]. Correlation analysis further established a statistically significant relationship between PLGF and both systolic and diastolic blood pressure, supporting the conclusions drawn by Zhang et al. [22].

The strengths of the study are that it is among the few that simultaneously evaluates a comprehensive set of demographic, clinical, laboratory, ultrasound, and outcome variables in relation to serum PLGF levels. The use of ELISA-based detection for PLGF, despite being research-focused, yielded results comparable to NICE (National Institute for Health and Care Excellence)-endorsed diagnostic tools. Additionally, the study offers robust evidence supporting the role of PLGF in predicting not only disease presence but also its severity and impact on maternal and neonatal outcomes.

However, several methodological limitations should be acknowledged. First, the use of ELISA kits, while accurate, limits clinical applicability due to longer turnaround times and a lack of suitability for rapid, point-of-care testing. Second, the study did not assess the cost-effectiveness of PLGF testing, which is essential for its integration into routine antenatal care, particularly in low-resource settings. Third, a control group of normotensive pregnant women was not included, which restricts the ability to determine the discriminatory accuracy of PLGF across the entire clinical spectrum. Fourth, the lack of blinding during clinical assessment could introduce observer bias, particularly if the assessors were aware of participants' PLGF status. Fifth, imminent signs were not objectively defined or standardized, and inter-observer variability in assessing clinical signs may have influenced outcome classification. Additionally, the study did not stratify outcomes based on gestational age at diagnosis of HDP or analyze the indications for early delivery, limiting causal inference regarding the role of PLGF in the timing of delivery or disease worsening. Lastly, the study was conducted at a single tertiary care center, which may affect generalizability to other populations or care settings.

## Conclusions

This study reinforces the significant correlation between serum PLGF levels and various demographic, clinical, laboratory, and ultrasound parameters in women with HDP, highlighting its potential as a reliable marker of disease severity. The association of low PLGF levels with adverse maternal and fetal outcomes further emphasizes its prognostic value. Based on our findings, early measurement of serum PLGF, especially in pregnancies with moderate to high gestosis scores, should be considered as part of routine antenatal care, ideally during the diabetic screening window. Integrating PLGF testing into clinical practice can enhance early detection, facilitate risk stratification, and enable personalized management strategies, ultimately improving maternal and perinatal outcomes.
